# Hibiscus, Rooibos, and Yerba Mate for Healthy Aging: A Review on the Attenuation of In Vitro and In Vivo Markers Related to Oxidative Stress, Glycoxidation, and Neurodegeneration

**DOI:** 10.3390/foods11121676

**Published:** 2022-06-07

**Authors:** Matheus Thomaz Nogueira Silva Lima, Eric Boulanger, Frédéric J. Tessier, Jacqueline Aparecida Takahashi

**Affiliations:** 1 Department of Food Science, Faculty of Pharmacy, Universidade Federal de Minas Gerais, Av. Antonio Carlos, 6627, Belo Horizonte 31270-901, Brazil; 2 U1167—RID—AGE—Facteurs de Risque et Déterminants Moléculaires des Maladies Liées au Vieillissement, Institut Pasteur de Lille, CHU Lille, Inserm, University Lille, F-59000 Lille, France; eric.boulanger@univ-lille.fr (E.B.); frederic.tessier@univ-lille.fr (F.J.T.); 3 Department of Chemistry, Exact Sciences Institute, Universidade Federal de Minas Gerais, Av. Antonio Carlos, 6627, Belo Horizonte 31270-901, Brazil; jat@qui.ufmg.br

**Keywords:** herbal teas, oxidative stress, glycoxidation, neurodegeneration, therapeutics

## Abstract

The world is currently undergoing a demographic change towards an increasing number of elderly citizens. Aging is characterized by a temporal decline in physiological capacity, and oxidative stress is a hallmark of aging and age-related disorders. Such an oxidative state is linked to a decrease in the effective mechanisms of cellular repair, the incidence of post-translational protein glycation, mitochondrial dysfunction, and neurodegeneration, just to name some of the markers contributing to the establishment of age-related reduction-oxidation, or redox, imbalance. Currently, there are no prescribed therapies to control oxidative stress; however, there are strategies to elevate antioxidant defenses and overcome related health challenges based on the adoption of nutritional therapies. It is well known that herbal teas such, as hibiscus, rooibos, and yerba mate, are important sources of antioxidants, able to prevent some oxidation-related stresses. These plants produce several bioactive metabolites, have a pleasant taste, and a long-lasting history as safe foods. This paper reviews the literature on hibiscus, rooibos, and yerba mate teas in the context of nutritional strategies for the attenuation of oxidative stress-related glycoxidation and neurodegeneration, and, here, Alzheimer’s Disease is approached as an example. The focus is given to mechanisms of glycation inhibition, as well as neuroprotective in vitro effects, and, in animal studies, to frame interest in these plants as nutraceutical agents related to current health concerns.

## 1. Introduction

People are living longer. Currently, emergent and developed countries are undergoing a demographic transition with an increasing number of elderly citizens. According to the World Health Organization (WHO), the total number of people over 60 years of age will account for 22% (2.1 billion) of the global population by 2050 [1]. The demographic aging process imposes a series of socioeconomic challenges owing to the elevated number of geriatric individuals affected by age-related diseases (e.g., metabolic dysfunction, neurodegenerative diseases, and cardiovascular diseases) [2]. These changes are linked to the temporal decline of physiological and cognitive capacities resulting from inefficient mechanisms of cellular repair (e.g., polymerase read-proofing activity), accumulation of non-functional cellular proteins (e.g., glycated proteins), genetic degradation (e.g., mutations), and mitochondrial dysfunction, which merge to define the molecular aging process. In addition to environmental factors, such as lifestyle (e.g., smoking, retirement, housing), exercise practice, and/or diet also play a role in aging acceleration [3,4].

An aging hallmark is the establishment of an oxidative stress state, which led to the formulation of the Oxidative Stress Theory of Aging [5]. This oxidative status coincides with the incidence of several chronic diseases, accounting for the accumulation of defective cellular apparatus [6]. At the biological level, distinct mechanisms create a fruitful environment for the aggravation of oxidative stress, including the accumulation of misfolded proteins (e.g., amyloid-beta peptides) [6], glycation [7], shifts in calcium homeostasis [8], dysfunction of the mitochondrial electron chain [9], and cellular signaling cascade disruptions [10]. Additionally, as cells undergo senescence, repair mechanisms become less efficient, including anti-oxidative (e.g., reduction in glutathione levels) [11], as well as anti-glycation barriers (e.g., glyoxalase system) [12].

A therapeutic strategy to elevate antioxidant defenses and overcome health challenges is the use of nutritional therapies [4]. Recently, there has been keen interest in healthier lifestyles, leading to the search for functional foods capable of reducing the deleterious effects of molecular aging [13]. This interest has been driven by many studies on natural products, mainly medicinal plants, which have provided insights into the protective effects of diverse phytochemicals [14,15]. Polyphenols are the most abundant bioactive compounds present in a variety of plant species [16]. When acquired through diet, polyphenols attenuate pathological processes related to oxidative stress, aging, and neurodegeneration, turning these metabolites into a commercial target for the food and pharmaceutical industries [17]. Other plant metabolites, such as alkaloids, have also been demonstrated to have a protective effect on the central nervous system, and have been seen as emerging alternative treatments for anxiety reduction, and as antidepressant drugs [18,19].

The data presented in this review highlight the bioactivity of herbal plants, such as hibiscus, rooibos, and yerba mate, in the attenuation of oxidative stress markers, glycoxidation, or neurodegeneration, both in vitro and in vivo (animal studies), which may contribute to aging slowdown [20,21,22,23,24,25]. Hibiscus, rooibos, and yerba mate teas are herein contextualized as alternative strategies for the attenuation of oxidative stress, to frame the current interest in these plants as nutraceutical agents. The essential mechanisms of age-related oxidative stress, particularly those associated with protein glycation, and neuropathologies are presented. The following sections describe the recent discoveries regarding the use of these plants in such pathological contexts. Finally, we address the perspectives and research gaps that remain to be filled.

## 2. Oxidative Stress Promoting Mechanism Related to Glycation and Neurodegeneration

### 2.1. Protein Glycation and Oxidative Stress

Post-translational protein modifications have a potential effect on molecular aging, cell dysfunction, and chronic disease development [26]. First introduced by the French biochemist Louis Camille Maillard, in 1912, the Maillard Reaction characterizes a series of non-enzymatic reactions between free amino acids, or protein-amino residues, and reducing carbohydrates, resulting in a group of heterogeneous and chemically stable neo-formed compounds called Advanced Glycation End-Products (AGEs) [27]. Clinical consequences were later discovered in the association between glycation and diabetes progression, cardiovascular implications, and vascular stiffness promotion [28,29]. Besides the spontaneous occurrence of glycation in vivo, thermal processing of foods also promotes the formation of AGEs [30]. Some dietary AGEs are of great interest to the food industry because of the appealing and sensorial aspects of food, such as flavor, aroma, and color, in addition to increasing the pool of glycation products in vivo [31,32]. In contrast, several AGEs (e.g., acrylamide, and carboxymethyl-lysine—CML) act as potential activators of inflammation, oxidative stress, or even disturbing gut epithelial homeostasis [33].

Some glycation pathways are described as aging-promoting mechanisms, such as the modifications in extracellular matrix proteins, the reduction of cellular connectivity, elasticity and tissue flexibility, the promotion of tissue loss of function, and oxidative stress/inflammation activation mediated by the specific interaction with the Receptor for Advanced Glycation End-products (RAGE) [34]. RAGE is a multi-ligand receptor part of the immunoglobulin superfamily that is involved in the initiation of innate pro-inflammatory responses and oxidative stress triggers [35,36]. Studies on AGE-RAGE axis activation linked increased expression of intracellular oxidative and pro-inflammatory signals, such as NADPH oxidase, and cytokines in renal tissue, respectively [37]. Another important implication of glycation in the cellular redox control is related to the loss of function of proteins related to redox control. Macromolecular changes have been reported in catalases [38], and glutathione [39], which are essential for redox homeostasis.

**Figure 1:**
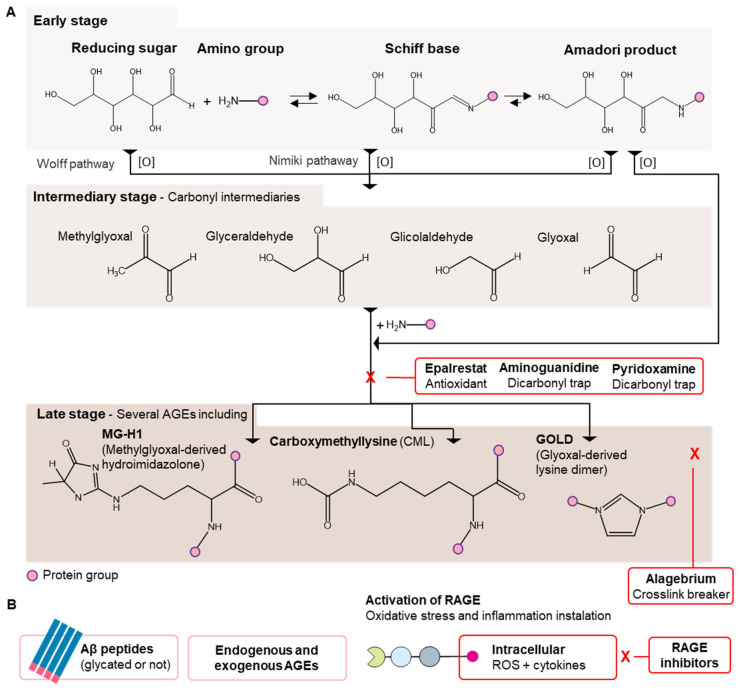
(**A**) Schematic representation of early stage of the Maillard Reaction and related oxidative pathways. The oxidative cleavage of Schiff base and Amadori Products results in the formation of reactive dicarbonyls. Dicarbonyls are also by-products of glucose autoxidation and glycolysis. Reactive dicarbonylic intermediaries are further involved in glycoxidation leading to the formation of AGEs [54]. Epalrestat, aminoguanidine, pyridoxamine, and alagebrium have been identified as mitigators of the Maillard Reaction, taking part in different steps. (**B**) The resulting AGEs in the latest phase of the Maillard reaction, both from endogenous and exogenous sources, have been identified as ligands and potential activators of RAGE. Such interaction leads to downstream activation of oxidative stress and inflammation. From the clinical perspective, a therapeutic strategy to mitigate the activation of RAGE, as well as the progression of Alzheimer’s Disease, has been focused on the development of RAGE inhibitors [55].

Together, glycation and oxidative stress lead to glycoxidation and downstream oxidative stress activation (Figure 1A) [40]. The early stage of the Maillard Reaction involves a series of reversible sugar-amino group rearrangements. The oxidative degradation of Schiff bases and Amadori Products, together with glucose autoxidation, and cellular metabolism (e.g., glycolysis), result in the generation of α-oxaldehydes (e.g., glyoxal, methylglyoxal) (Figure 1A). The formation of CML, one of the most discussed AGE in the pertinent literature, has been consistently demonstrated to increase under aerated conditions with different α-carbonyl precursors (glycolaldehyde and glyceraldehyde) [41,42].

Over recent decades, several compounds have been demonstrated to inhibit AGE formation, such as thiamine, and pyridoxamine [43], to inhibit the related redox imbalance during the early stages of the reactions, such as Epalrestat [44], or by breaking AGE cross-links, such as alagebrium [45] (Figure 1A). Aminoguanidine, a hydrazine derivative, was shown to be the most promising synthetic drug for AGE formation inhibition but severe countereffects associated with kidney damage diminished its clinical potential (Phase III) [46]. On the other hand, natural products have been used as a strategy to mitigate AGE formation [47]. Currently, the screening of natural compounds (e.g., polyphenols, polysaccharides, terpenoids, vitamins, and alkaloids) for new glycation inhibitors has gained attention because of the historically safe consumption profile of these molecules [47]. Natural products can act on any phase of the series of reactions on AGE formation pathways, from sugar-protein interaction blocking, to attenuating glycoxidation through trapping intermediates, including reactive dicarbonyls, or free radicals, and/or by breaking down formed AGE crosslinks [48,49].

Two main research fronts have been explored in the literature investigating natural products as inhibitors of protein glycation. First, is the use of natural products as nutraceuticals. Quercetin, for instance, has been demonstrated to trap methylglyoxal and glyoxal, thereby reducing subsequent AGE formation in vitro [50]. A similar effect was observed in mice that received oral quercetin supplementation. After 6 weeks of feeding, the group of animals in which quercetin was provided, together with methylglyoxal, had lower circulating levels of methylglyoxal and AGEs [51]. Second, food additives can inhibit glycation during food processing. In this second strategy, natural products are less explored because of the significant sensory changes in food (e.g., texture, color). Quercetin has been investigated in both cookies and bread models. In both foods, quercetin addition was able to consistently mitigate total AGE formation, but sensorial changes in dough elasticity were observed [52,53].

### 2.2. Oxidative Stress and Neurodegeneration: A Case of Alzheimer’s Disease

**Figure 2:**
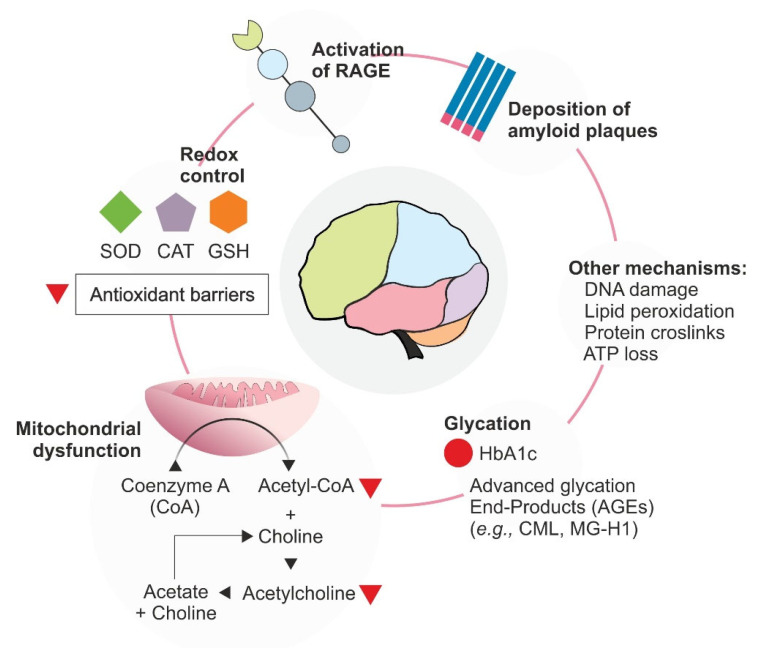
Schematic representation of some biological mechanisms in the interplay of glycation, neurodegeneration, and the progression of oxidative stress. Mechanisms in brain degeneration are highlighted. Both glycation and cellular degeneration are involved in the activation of local (i.e., brain) and systemic oxidative stress. The accumulation of dysfunctional mitochondria, DNA damage, lipid peroxidation, and/or energetic imbalance induces severe damage to cells. AGEs, as well as Aβ-peptides, have related stress activation on membrane-RAGE, which is a promiscuous receptor interacting with both ligands. CML: carboxymethyl-lysine; MG-H1: Nδ-(5-hydro-5-methyl-4-imidazolon-2-yl)ornithine.

The occurrence of exacerbated oxidative process is common in Alzheimer’s Disease and other chronic neurodegenerative disorders [56]. Many mechanisms are involved in the progression of oxidative stress in the brain (Figure 2). Gradual loss of neurons, motor impairment, and aggregation of proteins (e.g., TAU proteins, Aβ-peptides) characterize these diseases [57]. From the oxidative perspective, mitochondrial dysfunction is a common factor, since neurons are post-mitotic cells that demand large amounts of energy, use high levels of oxygen, and are inserted into an environment with lower antioxidant capacity, and are, therefore, more susceptible to damage and compos the Mitochondrial Hypothesis of aging [58]. This hypothesis identifies mitochondria, the cellular organelle responsible for energy production, as the main source of ROS (reactive-oxygen species), especially in energy-demanding organs, such as the brain [59]. In this context, excessive production of oxygen and reactive nitrogen species (NOS) promotes the oxidation of proteins, lipids, and nucleic acids, providing the emergence of pathologies, such as Alzheimer’s Disease [6].

Alzheimer’s Disease is a complex neurological dysfunction and the most prevalent form of dementia worldwide [60]. The origin of the disease can be either genetically determined, developed in early adulthood, or associated with aging [61]. The main pathophysiological feature of Alzheimer’s Disease is the deposition of extracellular fibrillar protein aggregates called amyloid-β peptides (Aβ) (a well-known RAGE ligand) and hyperphosphorylation of the TAU protein. These protein aggregates initiate neuroinflammatory processes, leading to neuronal death and synaptic communication failures [62]. They lead to a depletion of calcium in the endoplasmic reticulum, reducing the activity of GSH, and leading to the accumulation of ROS [63]. A clear association between the deposition of amyloid aggregates is related to the impairment of complexes IV and V of the mitochondrial electron transport chain, and increased permeability of mitochondrial membranes [64]. Mitochondrial impairment leads to a second molecular marker identified in Alzheimer’s Disease patients: the reduction in the synthesis of acetylcholine. Acetyl-CoA, an acetylcholine precursor, is synthesized in the mitochondrial matrix [65]. This neurophysiological landmark, described by the Cholinergic Hypothesis, is one of the main characteristics of the disease, and it may occur up to 30 years before the appearance of the first clear signs of the pathology [66]. Based on this physiological framework, the only commercially available drugs for the treatment of Alzheimer’s disease are inhibitors of the acetylcholinesterase enzyme that promotes the hydrolysis of acetylcholine, attenuating the effects of low levels of the neurotransmitter [67].

Glycation-related pathways are also involved in Alzheimer’s disease pathophysiology. Aβ-peptides have been identified as RAGE ligands [68] (Figure 1B). Aβ peptide crosslinks have been demonstrated to be associated with glycation, which was shown to modulate the interaction Aβ-RAGE, increasing the affinity of the glycated clusters to the receptor [69]. Furthermore, based on the in-situ analysis of neurons, Rudy et al. (2001) demonstrated the presence of CML in neurofibrillary tangles of patients with Alzheimer’s Disease [70].

## 3. Hibiscus, Rooibos, Yerba Mate as Sources of Natural Bioactive Compounds

As several pathological mechanisms hold an oxidative stress factor, some challenges have been encountered in limiting the real perspectives on antioxidant therapy application. There is a lack of consensus in the scientific literature regarding the effectiveness of antioxidant therapies. Some reasons have been recently pointed out concerning the definition of the real extension of oxidative stress as a disease-promoting factor, added to underestimation of the related pathways the therapies could engage. Yet, there is no definition of effective doses for in vivo effect [71]. The simple scavenging of radicals would be a simplistic use to fulfil gaps in the potential of plant bioactive compounds as potential antioxidant molecules. An overview should be done on related mechanisms in the activation of physiological pathways, such as the SOD (Superoxide dismutase) system, CAT (Catalase), or the downregulation of pro-inflammatory cytokines, such as TNF-α, related to the downstream activation of oxidative stress. Flavonoids from Chinese medicinal herbs have been demonstrated to modulate such pathways, contributing to the regulation of oxidative stress in mice brains [72].

The in vitro antioxidative effect of plant bioactive compounds has been largely demonstrated. When it comes to the epidemiological approach, studies on the consumption of plant-based diets rich in polyphenols have been published since early 1990. A follow-up study (5 years) with 805 individuals in the Netherlands indicated the daily consumption of 259 mg of flavonoids, mainly from teas (61%), was inversely proportional to the risk of coronary heart diseases. Such a relationship was later demonstrated in other populations, but a limiting factor in this issue has recently been raised from metadata analysis, which demonstrated that the great variation in terms of bioactive compound intake across different studies limits the effects of translation from in vitro to in vivo trials [73].

Many plant species exhibit significant neuroprotective in vitro and in vivo activities [74]. These plants contain multiple molecules, capable of working through different mechanisms of action, which could benefit the search for therapeutics for complex neurodegenerative disorders. Flavonoids, for instance, are ubiquitously distributed plant constituents, with more than 6000 structures already identified. These polyphenolic compounds are notorious antioxidants that must be acquired from the diet, as they are not biosynthesized by the human body [75]. Flavonoids have been reported to be effective neuroprotective agents with several advantages, such as safety, good pharmacokinetic flow, capacity to penetrate the blood-brain barrier, and cost-effectiveness [76]. Alkaloids are another class of ubiquitous bioactive natural products. These nitrogen-containing natural products have a wide array of chemical structures, that have long been known for their therapeutic activities, especially by stimulating the central nervous system in humans [77]. Alkaloids also act as anti-cancer and neuroprotective agents [78,79], as well as in the cardiovascular system [80]. The main alkaloid present in food sources is caffeine, a dimethylxanthine present in coffee and cocoa (Table 1) [81].

**Figure 3:**
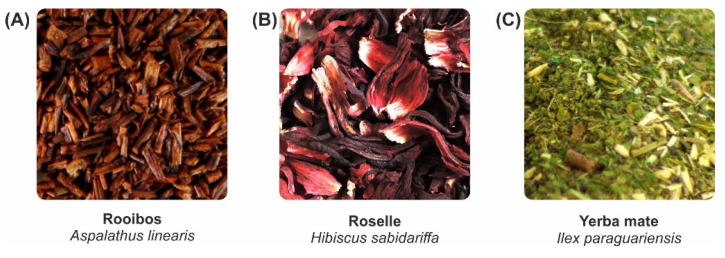
Commercial (**A**) rooibos, (**B**) hibiscus, and (**C**) green yerba mate dry herbal teas.

Flavonoids and alkaloids are present in a wide variety of foods, including herbs that have remarkable therapeutic properties and have long been used in tea preparations. Teas are the most consumed plant-based beverages with a high-value market around the world, expected to reach $318 billion by 2025 [82,83]. The tea market is mainly driven by black, oolong, and green tea products derived from Camellia sinensis [84]. Comprehensive reviews have been published on the phytochemical composition of fermented and non-fermented C. sinensis leaves, which are composed of phenolic compounds (flavan-3-ols, epicatechins, catechins), and alkaloids, such as theobromine [85]. These plant metabolites have been associated with positive health effects, including antioxidant, anti-inflammatory, cardioprotective, and neuroprotective [86]. In response to market demand, the food industry is urging the development of new products with functional and health claims, leading other herbal infusions to gain public attention in European, American, and Asian markets [87]. Hibiscus (Hibiscus sp.), rooibos (Aspalathus linearis), and yerba mate (Ilex paraguariensis) are important examples of herbal plants (Figure 3) that are mostly found in African and South American local markets and have been traditionally used in the production of flavorful non-alcoholic beverages [88,89,90]. These plants have been demonstrated to have important health effects, such as as anti-cancer (in vitro) [91], anti-diabetes (in vivo) [92], or anti-inflammatory (in vivo) [93] effects. These health benefits play a role as commercial boosters for increasing market demand for new plant-based products with functional properties. The hibiscus market, for instance, is projected to grow 7% by 2027. During 2021–2027, the yerba mate market is estimated to increase by almost 5%, while the rooibos market is mainly held by South African farmers with 7000 hectares and 15000 tons produced yearly [94,95,96]. These crops are alternatives to C. sinensis tea, the market of which has shrunk over the last years due to adverse climate conditions [97]. Therefore, investing in hibiscus, rooibos, and yerba mate markets may contribute to local crop expansion, more sustainable and biodiverse agricultural development, and the reduction of local inequalities among small-scale farmers.

The Hibiscus genus is native to Africa, with distribution between the Middle East, Asia, and Latin America, and has great taxonomic diversity with more than 300 species cataloged between the tropics and subtropics [88]. The main species of hibiscus intended for human consumption is H. sabdariffa (Figure 3), popularly known as roselle, and H. rosa-sinensis, the Chinese mellow. In African countries, such as Egypt and Nigeria, extracts of H. sabdariffa are added to gelatin and fruit juices to improve the nutritional value and attractiveness of foods [98]. The nutritional value of hibiscus petals is corroborated by the presence of vitamins A and E, ascorbic acid (vitamin C), calcium, and iron [88]. The phytochemical profile of hibiscus extracts also comprises tocopherol and linoleic acid, organic acids (malate, oxalate, citric, and hibiscus acid); phenolic acids (caffeic and chlorogenic acids), and other phenolic compounds, such as flavonoids and anthocyanins [99]. The first anthocyanin isolated from aqueous extracts of hibiscus was hibiscin (delphinidin-3-sambubioside), which corresponds to 71% of the anthocyanin content in hibiscus extracts [100]. This metabolite has long been known to participate in the modulation of mitochondrial ROS production and the induction of apoptosis in human leukemic cells [101]. Furthermore, it can mediate inflammatory processes by reducing intracellular inflammatory signals, such as IL-6 and TNF-α [102]. The related effects of hibiscin and other frequently investigated phytochemicals in hibiscus, rooibos, and yerba mate are presented in Table 1.

Rooibos (Figure 3) is a common name for the shrubby legume from the South African species A. linearis. Rooibos is prepared from either leaves or stems, and consumption of it has been increasing because of its health benefits and caffeine-free composition, compared to other teas [103]. In addition, this plant has a unique flavonoid profile including dihydrochalcone aspalathin in both fermented and non-fermented rooibos, which has been demonstrated to help with type-2 diabetes slowdown [104]. Non-exclusive polyphenols, such as orientin, isoorientin, isoquercitrin, rutin, and quercetin, have also been described in rooibos [105].

Yerba mate (Figure 3) tea results from I. paraguariensis leaf infusions, a plant originally from South America, that is endemic to Brazil, Argentina, Paraguay, and Uruguay where the beverage is traditionally consumed [106]. Yerba mate infusions are either consumed from green or toasted leaves. According to scientific reports, the leaves have been demonstrated to be predominantly composed of chlorogenic acids and xanthine derivatives, such as caffeine and theobromine, which have antioxidant (in vitro) [107], as well as hepatoprotective (in vivo) [108], antimicrobial [109], and anti-cancer (in vitro) effects [110].

**Table 1:** Reported bioactive natural products in rooibos, hibiscus, and/or yerba mate extracts.

Experimental Condition	Compound [Class]	Chemical Structure	Associated Bioactivity [Model]	Effect
In vitro	Caffeic acid [Phenolic compound]	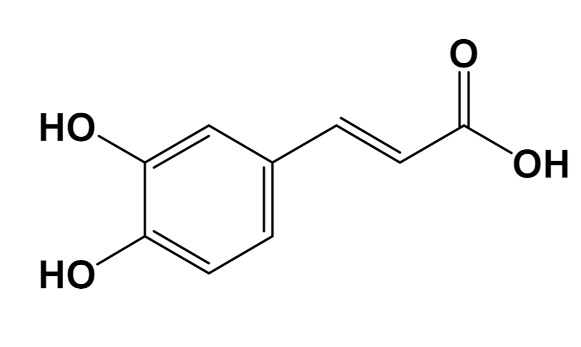	Anti-glycation [Fluorescence 370/440 nm]	AGE formation Control: 180% Caffeic acid (0.2 mM): 80% [111]
	Epicatechin [Polyphenol]	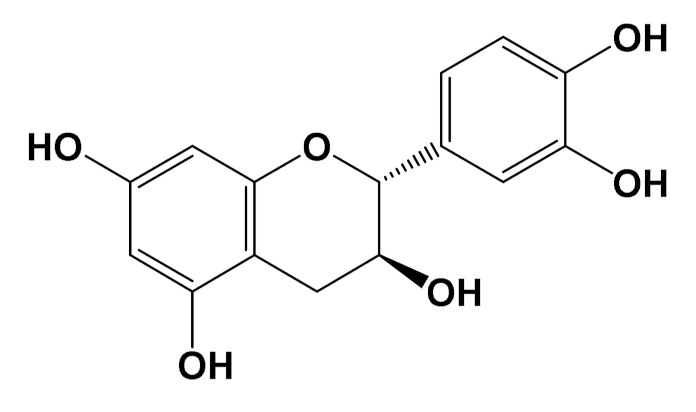	Neuroprotective [SHSY5Y cells]	Parkin expression Rotenone (1 μM): 110 (a.u) Rotenone + Epicatechin (10 μM): 60 (a.u.) [112]
	Hibiscin [Polyphenol]	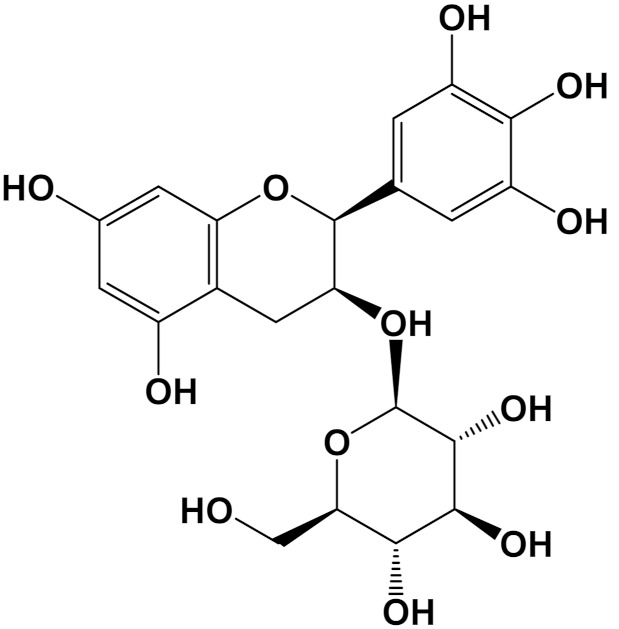	Anti-inflammatory [RAW264.7 macrophage cells]	IL-6 expression Control (LPS 1 mg/kg): 1750 pg/mL LPS + Hibiscin (15 µM/kg): 750 pg/mL [102]
	Quercetin [Polyphenol]	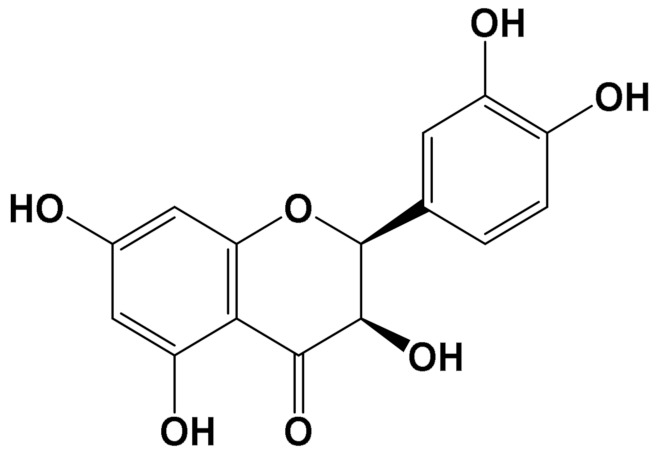	Attenuation of mitophagy [Primary microglia]	MitoSox Control (LPS (100 ng/mL): 28 (a.u.) LPS + Quercetin (30 μM): 8 (a.u.) [113]
	Quinic acid [Polyol]	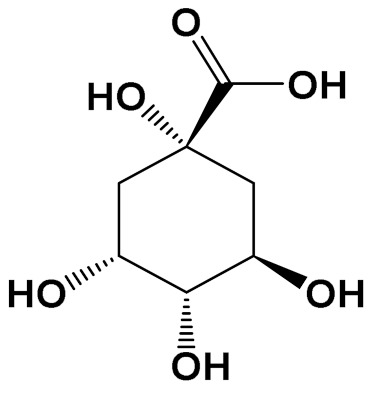	Photoprotective [HaCaT keratinocytes]	UVB irradiation-induced ROS generation Control: 2000 (a.u.) Quinic acid: (10 uM): 1500 (a.u.) [114]
	Theobromine [Methylxanthine]	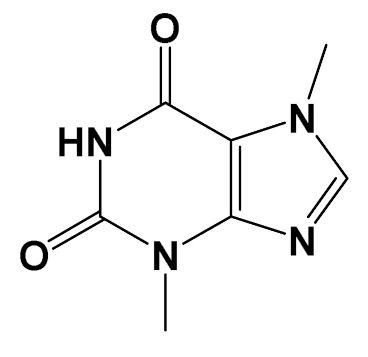	Adipogenesis attenuation [SGBS cells]	Adipogenic differentiation Control: 100% Teobromine (100 µg/mL): 60% [115]
In vivo	Acteoside [Polyphenol]	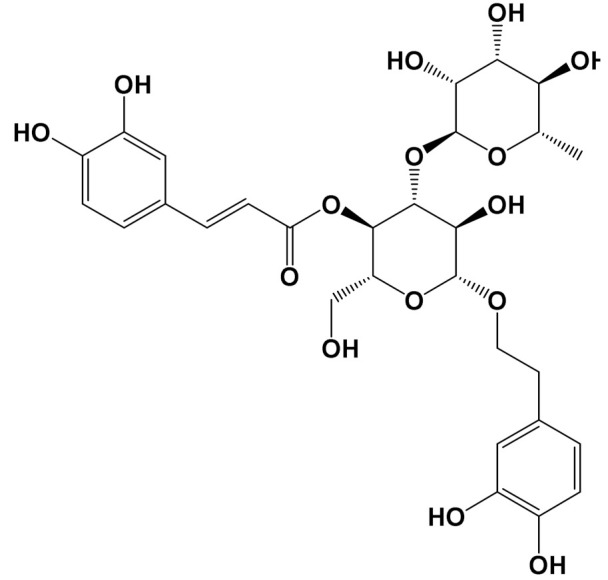	Glucose metabolism [Male Wistar albino rats]	Blood glucose Control: 318 mg/dL Acteoside (40 mg/kg): 75 mg/dL (Oral) [116]
	Aspalathin [Polyphenol]	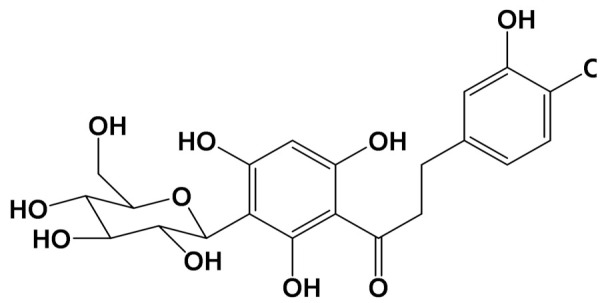	Nephroprotective [Male C57BL/6 mice]	Creatinine levels Control: 0.47 mg/dL Aspalathin (1.00 mg/kg): 0.25 mg/dL (Intravenous injection) [117]
	Caffeine [Methylxanthine]	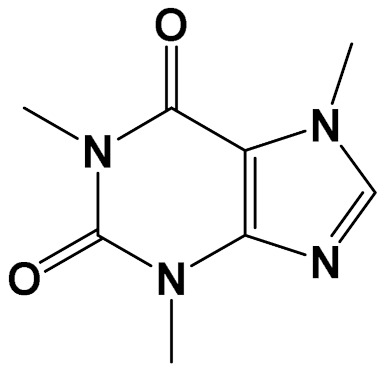	Anti-AChE [Male albino rats]	AChE activity Control: 7 µmol/min/g Caffeine (20 mg/kg/day): 2 µmol/min/g (Oral) [118]
	Chlorogenic acid [Polyphenol]	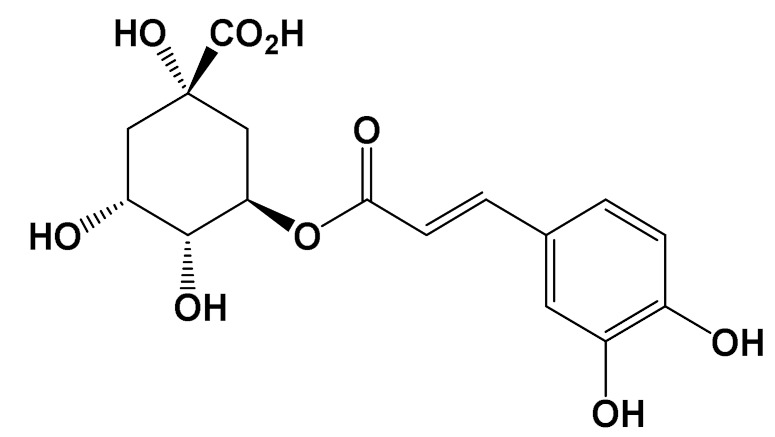	Neuroprotective [Swiss albino mice]	Mitochondrial damage Control (MPTP): 3 nM Chlorogenic acid + MPTP: 5 nM (Oral) [119]

SGBS: human Simpson-Golabi-Behmel syndrome (SGBS) preadipocyte cell; SHSY5y: human neuroblastoma cell line; RAW262.7: murine monocyte/macrophage-like cells.

## 4. The Potential of Hibiscus, Rooibos, and Yerba Mate in Glycoxidation and Neurodegeneration Attenuation

The well-known food safety associated with the consumption of hibiscus, rooibos, or yerba mate, and the acceptance of these herbs linked to pleasant taste, have elicited great interest in defining their nutraceutical potential. So far, no acute toxic effects have been inferred from the consumption of hibiscus or yerba mate extracts, based on short or long-term experiments in rodents, neither from histochemical tissue assays nor biochemical serum analysis [120,121]. On the other hand, cases of acute hepatitis in South Africa and France have been recently described to be potentially associated with rooibos consumption, but no clear link has been stated in both case reports [122,123]. Recent discoveries have been made, both in vitro and in vivo (animal models), on the effects of these plants as antioxidants, for the prevention of AGE formation, and neuroprotective effects. The major bibliography addressing these plants relies on the effects of crude aqueous extracts in a way that mimics the traditional methods of preparation. With the increasing understanding of the diverse mechanisms leading to elevated ROS levels, more significant biomarkers have been explored in the literature to draw conclusions on the effects of phytochemicals in this context. Table 2 summarizes some major targets investigated on hibiscus, rooibos, and yerba mate-related literature associated with oxidative stress, extrapolating ROS production measures. Estimating oxidative constraints may range from the direct detection of oxidative species to their impacts as oxidized lipids. However, while the expression of CAT, SOD, or MAO (Monoamine oxidase) is well explored in the context of the consumption of these herbal drinks, other markers of oxidative stress remain to be investigated as glycation products (e.g., pentosidine, carboxyethyl-lysine, CML) [124,125]. Due to increasing clinical interest and the significance of the causal role of redox modifications related to glycoxidation and neuro-oxidation, the use of herbal teas in recent years is highlighted as an attenuation strategy.

### 4.1. Antioxidant and Anti-Glycation Effects of Hibiscus, Rooibos, and Yerba Mate

The brain, the liver and other organs appear to be sensitive to oxidative stress [138,139,140]. Some body of work has addressed the potential of hibiscus, rooibos, and yerba mate crude extracts in the mitigation of ROS production, as well as anti-glycation, both in vitro (Table 3) and in vivo (Table 4), approaching major biomarkers as glutathione, SOD, CAT, and the formation of autofluorescent AGEs. In vitro studies on neuroblastoma cell culture (SH-SY5Y) demonstrated that hibiscus ethanolic extracts (100 µg/mL) reduced ROS production, and more significantly lipid peroxidation, when compared to cells exposed to H_2_O_2_ stress, which is supposed to contribute to cell membrane lipid layer maintenance [141]. Under in vivo conditions, such antioxidant potential was translated as increased engagement of CAT and SOD enzymes in the brain of diabetic male Sprague-Dawley rats who orally received 25 mg/kg body weight of hibiscus aqueous extract [142].

The effect of rooibos was similar over SOD and CAT, as observed in immobilization-induced oxidative stress Sprague-Dawley animals receiving a supplement of rooibos, in a 4-week study. The intake of rooibos aqueous extract (10 mg/mL) was demonstrated to result in greater activity of both enzymes in comparison to animals under stress but not receiving rooibos supplementation [143]. In consequence, in this same study, rooibos was associated with lower brain lipid oxidation. Rooibos is considered to act over DAF-16/FOXO signaling pathway, which mediates SOD, CAT, and GST levels, modulating life span [21].

**Table 3:** In vitro antioxidant and anti-glycation effects of rooibos, hibiscus, yerba mate extracts.

Assay	Species [Extract]	Measure	Dose or EC_50_	Reference
Antioxidant	*H. sabdariffa* [Ethanolic]	Lipid peroxidation (SH-SY5Y cells)	Control: 800% Extract (100 µg/mL): 300%	[141]
		ROS production (SH-SY5Y cells)	Control: 130% Extract (100 µg/mL): 100%	
	*H. sabdariffa* [Methanolic]	Malondialdehyde	EC_50_ 22 μg/mL	[144]
		Monoamine Oxidase	EC_50_ 44 μg/mL	
		ATPase activity	EC_50_ 22 μg/mL	
Anti-glycoxidation	*A. linearis* [Aqueous]	AGE formation inhibition (Fluorescence 340/420 nm) Glucose in BSA system	Control (aminoguanidine): 45% Green extract (200 μg/mL): 45% Fermented extract (200 μg/mL): 55%	[145]
	*H. rosa-sinensis* [Aqueous]	AGE formation inhibition (Fluorescence 340/420 nm) Fructose in BSA system	Control (Aminoguanidine): IC_50_ 6 μg/mL Extract: IC_50_ 67 μg/mL	[146]
	*I. paraguariensis* [Aqueous]	AGE formation inhibition (Fluorescence 340/420 nm) Fructose in BSA system	Control (Fructose): 4000 a.u. Extract (2.5 µg/mL): 3000 a.u.	[147]
		AGE formation inhibition (Fluorescence 340/420 nm) Methylglyoxal in BSA system	Control (green tea): 65 a.u. Extract (20 µg/mL): 42 a.u.	[20]

BSA: bovine serum albumin.

Oxidative stress and inflammation are interconnected mechanisms that play roles in chronic disease progression [148]. Hibiscus was also demonstrated to attenuate the effect of markers on the interface between oxidative stress and inflammation. COX-2 is a mediator in inflammatory action, while monoamine oxidase (MAO) plays a major role in the outer mitochondrial membrane, regulating the metabolism of monoaminergic neurotransmitters [129,134]. Compelling evidence involves both biomarkers in the progression of ROS-related inflammation in major metabolic disorders [149,150]. Oboh et al. (2018) reported that roselle methanolic extract reduced MAO expression in vitro (EC_50_ = 43.69 µg/mL), while diabetic Wistar albino mice had decreased COX-2 activity toward the inversion of oxidative stress [25].

Glutathione (GSH) is a powerful mechanism in animal cell redox control [151]. It has been demonstrated that aging neurons have lower levels of the reduced form (GSH) which is converted into the oxidized version (GSSG) [152]. Oral supplementation of rooibos (10 mg/mL) and yerba mate (200 mg/mL) extracts showed effects on the increase of the GSH/GSSH ratio. Such behavior attributed to yerba mate was also observed in synaptosomal/mitochondrial P2 fractions [153], as well as in brain homogenates of chronic immobilized rats [154], which suggests that synaptosomal cells are key in GSH control in rats.

Rooibos, hibiscus, and yerba mate provide an important phytochemical repertoire with anti-glycoxidation activity. Reactive saccharides, such as glucose, fructose, and ribose, as well as carbonyl compounds, such as glyoxal, and methylglyoxal, have been described as important precursors of AGEs [41]. Therefore, in the search for anti-glycation molecules, different glycation precursors are investigated. Several glycation derivatives, including protein cross-links, are auto-fluorescent and can be detected at excitation/emission wavelengths of 335/385 nm, for total AGE estimation, and 485/520 nm for cross-link estimation [155,156]. This characteristic is explored in vitro for bioassays on the inhibition of AGE formation. Caffeic and chlorogenic acid were found to be major components in *I. paraguariensis* extracts. Along with the study of the inhibition of AGEs, based on fluorescence measures, caffeic acid showed the most significant effect (90%) in a methylglyoxal-BSA system compared to aminoguanidine (60%) control [157]. Chlorogenic acid, on the other hand, showed similar EC_50_ to aminoguanidine, 10 mM and 8 mM, respectively, in fructose/inhibition in the ovalbumin system [158]. When it comes to the crude extracts of yerba mate (2.5 µg/mL), a reduction of 25% occurred in the formation of fluorescent AGEs [147], while rooibos non-fermented extract (200 µg/mL) was shown to limit fluorescence up to 45%, equivalent to the aminoguanidine control [146]. In vivo, elevated glucose levels in diabetic patients have been correlated to the occurrence of glycated hemoglobin [159]. These polyphenols, as well as rutin and quercetin (also part of the phytochemical composition of these plants), act mainly by the inhibition of Amadori product formation in the early stage of the Maillard Reaction [160,161]. In addition, they may also contribute to glucose homeostasis by insulin resistance reduction, decreasing circulating AGEs, and lipid peroxidation in diabetic rats. Hibiscus tisane was demonstrated to play a role in circulating glucose and AGE reduction, while reducing the incidence of glycated hemoglobin [162].

**Table 4:** In vivo antioxidant and anti-glycation effects of rooibos, hibiscus, yerba mate extracts.

Target Effect/Organ	Species [Extract]	Concentration	Animal Model	Measure	Effect	Tendency	Reference
Antioxidant/Brain	*A. linearis* [Aqueous]	1 g/100 mL	Immobilization-induced oxidative stress Sprague Dawley rats	CAT	Control (Stress): 2 unit/mg Extract: 3 unit/mg	↑	[143]
				FFA	Control (Stress): 700 µg/mL Extract: 650 µg/mL	↓	
				GSH/GSSG	Control (Stress): 7.5 Extract: 9	↑	
				HIAA	Control (Stress): 400 mg/g tissue Extract: 350 mg/g tissue	↓	
				Lipid peroxidation	Control (Stress): 50 nmol/g tissue Extract: 40 nmol/g tissue	↓	
				SOD	Control (Stress): 1 unit/mg Extract: 1.7 unit/mg	↑	
	*H. rosa-sinensis* [Aqueous]	25 mg/kg body weight	STZ induced diabetic Male Sprague-Dawley	CAT	Control (Diabetic): 5 U/mg Extract: 10 U/mg	↑	[142]
				SOD	Control (Diabetic): 7 U/mg Extract: 15 U/mg	↑	
	*H. sabdariffa* [Aqueous]	200 mg/kg body weight	Male Swiss albino mice	MDA	Control (STZ): 3 nmol/g White hibiscus extract: 0.5 nmol/g Red hibiscus extract: 0.5 nmol/g	↓	[25]
				MPO	Control (STZ): 75 µg/mg tissue White hibiscus extract: 20 µg/mg tissue Red hibiscus extract: 20 µg/mg tissue	↓	
				Cox-2	Control (STZ): 4 (fold change) White hibiscus extract: 1 (fold change) Red hibiscus extract: 1 (fold change)	↓	
	*H. sabdariffa* [Ethanolic]	500 mg/kg body weight	Cypermethrin oxidative stress male mice (*Mus musculus*)	AChE	Control (Cypermethrin): 0.5 µmol/min/mg Extract: 2.5 µmol/min/mg	↓	[163]
				CAT	Control (Cypermethrin): 0.04 µmol/min/mg Extract: 0.06 µmol/min/mg	↓	
				H_2_O_2_	Control (Cypermethrin): 1.2 µmol/mg Extract: 0.3 µmol/mg	↓	
				MDA	Control (Cypermethrin): 2 µmol/mg Extract: 0.5 µmol/mg	↓	
	*I. paraguariensis* [Aqueous]	200 mg/mL	Chronic immobilization stress male Wistar rats	GSH/GSSG	Control: 0.48 Extract: 0.50	→	[154]
				Lipid peroxidation	Control: 2.1 TBA/mg Extract: 1.3 TBA/mg	↓	
		200 mg/mL	Male Wistar rats	GSH/GSSG	Control: 4.7 Extract: 16.6	↑	[153]
				Lipid peroxidation	Control: 1.3 MDA eq/mg Extract: 0.3 MDA eq/mg	↓	
		50 mg/kg BW	PTZ-induced seizure male Wistar rats	CAT	Control (PTZ): 5 mmol/min/mg Extract: 9 mmol/min/mg	↑	[164]
				SOD	Control (PTZ): 15.50 U/mg Extract: 23 U/mg	↑	
				Sulfhydryl protein	Control (PTZ): 0.09 nmol DTNB/mg Extract: 0.31 nmol DTNB/mg	↑	
Anti-glycoxidation	*H. rosa-sinensis* [Ethanolic]	25 mg/kg BW	STZ induced diabetic Male Sprague-Dawley	Glycated hemoglobin	Control: 13% Extract: 6%	↓	[142]
	*H. sabdariffa* [Methanolic]	200 mg/kg BW	STZ induced diabetic Male Sprague-Dawley	Serum glucose	Diabetic control: 400 mg/dL Extract: 100 mg/dL	↓	[162]
				AGE levels	Diabetic control: 4.5 mg/mL Extract: 3 mg/dL	↓	

STZ: streptozotocin.

### 4.2. Neuroprotective Effects of Hibiscus, Rooibos, and Yerba Mate

Several studies have shown that plant metabolites, such as flavonoids, anthocyanins, and phenolic acids, are active components with neuroprotective properties [165]. Complementary in vitro and in vivo assays demonstrated that *H. sabdariffa* led to the inhibition of AChE and butyrylcholinesterase (BChE), both related to the hydrolysis of acetylcholine [25,144] (Table 5). So far, more prolific research on this issue is found over hibiscus tisane. Table 5 exemplifies the investigation of different organic extractions of *H. sabdariffa*. Data from PC12 cells, a cell model for neural crest neuroblastic cells, demonstrated that hibiscus ethanolic extract (60 µg/mL) allowed the reduction of apoptotic cell counts [166].

When it comes to in vivo assays (Table 6), a diet enriched with hibiscus anthocyanins was able to downregulate several aspects of Alzheimer’s Disease, such as neuroinflammation. The aggregation of Aβ-peptides in the brain is a source of oxidative stress and was demonstrated to lead to lipid peroxidation [167]. In addition, Aβ-peptides play a role as a RAGE ligand, which account for a factor in oxidative stress in astrocytes and cerebral endothelial cells, as reported by [168]. In non-transgenic Alzheimer’s Disease model mice, Aβ-42 accumulation was reduced following γ-secretase, APH1a, and BACE1 activity [25]. *C. elegans* is a simple nematode, with an approximately 83% genome similar to humans, which means it is extremely useful in human physiological studies [169]. Yerba mate extract was able to downgrade neuro-oxidative biomarkers, such as Aβ-42 expression and ROS levels, in *C. elegans*. Most importantly, such effects were correlated to increased worm lifespan, suggesting that yerba mate extract can help to slow down aging [170].

In addition to these findings, some data on animal behavior shed light on the neuroprotective effects of hibiscus and yerba mate teas. Some strategies are used for neuronal damage perception, such as behavioral assay associated with anxiety-related, cognitive and spatial learning, and aversive memory. Respectively, elevated plus maze, Morris water test, and step-down avoidance tasks are behavioral tests able to estimate such cognitive impacts [171,172,173]. The Morris water maze test evaluates mice spatial reference. Regarding this issue, El-Shiekh et al. (2020) demonstrated that hibiscus flower extracts (both red and white flowers) (200 mg/kg) were able to restore mice spatial capacities compared to STZ-induced Alzheimer’s Disease model mice. Hibiscus was suggested to attenuate neuroinflammation and amyloidogenesis in the treated animals. In anxiety and memory assessment, it has been demonstrated that yerba mate hydroethanolic extract (300 mg/kg body weight) increased anxiolytic-like behavior in mice, which was suggested to be due to the bioactivity of yerba mate extracts over the cholinergic system, together with the levels of caffeine in this plant. On the other hand, scopolamine-induced deficit was prevented by ilex extract [22].

**Table 6:** In vivo neuroprotective effects of rooibos, hibiscus, yerba mate extracts.

Species [Extract]	Concentration	Animal Model	Measure	Effect	Tendency	Reference
*A. linearis* [Aqueous]	100 mg/mL	Zebrafish larvae	Monoamine oxidase	Control (Clorgyline): 100% Extract: 60%	↓	[174]
			Cell viability	Control: 100% Extract: 40%	↓	
	12.5 µg/mL	Zebrafish larvae	ROS production	Control: 600% (120 min) Extract: 200% (120 min)	↓	
*H. sabdariffa* [Aqueous]	200 mg/kg BW	Male Swiss albino mice	Moris water test	Control (STZ): 20 sExtract: 30 s	↑	[25]
			BACE1	Control (STZ): 5 (fold change) White hibiscus extract: 2 (fold change) Red hibiscus extract: 2 (fold change)	↓	
			Aβ-42	Control (STZ): 250 mg/mg tissue White hibiscus extract: 100 mg/mg tissue Red hibiscus extract: 100 mg/mg tissue	↓	
			γ-secretase	Control (STZ): 3.5 (fold change) White hibiscus extract: 1 (fold change) Red hibiscus extract: 1 (fold change)	↓	
*H. sabdariffa* [Ethanolic]	500 mg/kg BW	Swiss albino mice	AChE activity	Control (Scopolamin): 44 nM/min/g tissue Extract: 33 nM/min/g tissue	↓	[175]
*I. paraguariensis* [Aqueous]	10.5 mg/L	*Caenorhabditis elegans*	Aluminum induced oxidative stress	Control: 0.6 µM/h/mg Extract: 0.4 µM/h/mg	↓	[176]
*I. paraguariensis* [Ethanolic]	4 mg/mL	*C. elegans*	Aβ-42 expression	Control: 1 a.u. Extract: 0.6 a.u.	↓	[170]
			AChE activity	Control: 100% Extract: 50%	↓	
			Lifespan	Control: 15 days Extract: 17 days	↑	
			ROS production	Control: 100% Extract: 50%	↓	
	500 mg/kg	Male C57Bl/6 mice	Catalepsy	Control (reserpine): 120 s Extract: 60 s	↓	[177]
	300 mg/kg BW	Male Swiss mice	Elevated Plus Maze	Control: 17% Extract:40%	↑	[178]
			AChE	Control: 4.5 mmol/min/mg Extract: 8.0 mmol/min/mg	↑	
			Step-down avoidance task	Control: 170 s Extract: 70 s	↓	

## 5. Conclusions

Over the past few years, the mitigation of oxidative stress has gained much importance due to its implications on several modern diseases related to age progression, glycoxidation, and aging. The lack of effective treatments for neurodegenerative diseases or strategies that could prevent the onset of age-related diseases, for instance, encourages the keen interest of pharmaceutical and food industries in the search for products with improved bioactivity. Attenuating the implications of glycation on aging has caught attention as a potential anti-aging strategy.

Plants have important applications in the food industry and as nutraceuticals potentially minimizing the negative consequences of oxidative stress. Plants have a repertoire of phytochemicals that can be explored as food supplements, attenuating the progression of diseases, or as food additives, preventing the formation of neo-formed compounds with deleterious effects on human health. Rooibos, hibiscus, and yerba mate tisanes have been demonstrated to contribute positively to the reduction of oxidative stress, inhibition of glycoxidation, and promotion of neuronal oxidative protection, both in vitro and in vivo. Investigating these plants has important market impacts. The associated health claims and industries may be encouraged by the appealing taste that these plants present, great market acceptance, and lower health risks due to their long use as foods. Further characterization of the mechanisms of action on glycation inhibition or neuroprotection remains to be investigated, together with the identification of target compounds contributing to such effects. In addition, further investigation may benefit both the pharmaceutical and the food industries.

From the clinical point of view, it would be of great importance to approach the translation of described in vitro and pre-clinal results into human physiology. Such investigation may answer questions on the real effectiveness of these plants as nutraceuticals, as well as on the definition of optimal dose, and digestibility. From the food industry perspective, it would be of considerable importance to investigate the stability of key-bioactive compounds during food processing. Therefore, new insights are required to expand the biotechnological uses of these plants to help improve the human aging process. Lastly, besides the scope of this article, it is important to highlight that investing in and boosting hibiscus, rooibos, and yerba mate markets may contribute to local crop expansion, more sustainable development, the development of technological methods of production, and the reduction of local inequalities.

## Figures and Tables

**Table 2:** Biomarkers related to oxidative stress, glycoxidation, and neurodegeneration.

Physiological Target	Biomarker	Pathological Implication	Reference
Neurodegeneration	Aβ-peptides (Amyloid beta-peptide)	Aβ cerebral deposition increases with AD progression	[126]
	AChE (Acetylcholinesterase)	Participates in acetylcholine level decline in the genesis of AD	
	γ-secretase (Gamma secretase)	γ-secretase participates in Aβ-protein processing	
Glycoxidation	Fluorescence (355/460 nm)	Marker of AGE occurrence (e.g., skin)	[127]
Oxidative stress	CAT (Catalase)	Takes part in cellular oxidative stress mitigation	[128]
	COX-2 (Cyclooxygenase-2)	Inflammation and inflammation mediator	[129]
	GSH/GSSG (Reduced glutathione/oxidized glutathione ratio)	Redox balance indicator	[130]
	H_2_O_2_ (Hydrogen peroxide)	Mitochondrial dysfunction	[131]
	LDH (Lactate dehydrogenase)	Energy metabolism and cell senescence control	[132]
	Lipid peroxidation	Cellular lipid integrity biomarker	[133]
	MAO-A (Monoamine oxidase A)	Regulates amine metabolism, especially important for neurophysiology, associated with anxiety or depression studies	[134]
	MPO (Myeloperoxidase)	MPO is mostly produced by immune cells, especially neutrophils, being involved with both inflammation and oxidative stress	[135]
	SOD (Superoxide dismutase)	Plays a role in oxidative stress and cell injury indication	[136]
	HIAA (5-Hydroxyindoleacetic acid)	Product of serotonin metabolism pathway used as a biomarker of neurological injury	[137]

**Table 5:** In vitro neuroprotective effect of aqueous, ethanolic, and methanolic H. sabdariffa extracts.

Extract	Measure	Dose or EC50	Reference
Aqueous	AChE inhibition	Control (galantamine): IC_50_ 7 μg/mL White hibiscus extract: IC_50_ 123 μg/mL Red hibiscus extract: IC_50_ 106 μg/mL	[25]
Ethanolic	PC12 cells Inhibition of cell apoptosis	Control (SGD): 65 apoptotic cells Extract (60 µg/mL): 30 apoptotic cells	[166]
Methanolic	AChE inhibition	IC_50_ 46.96 μg/mL	[144]
	BChE inhibition	EC_50_ 40.38 μg/mL	
